# RSPO3 is a novel contraction-inducible factor identified in an “in vitro exercise model” using primary human myotubes

**DOI:** 10.1038/s41598-022-18190-z

**Published:** 2022-08-22

**Authors:** Tadahisa Takahashi, Yuqing Li, Weijian Chen, Mazvita R. Nyasha, Kazumi Ogawa, Kazuaki Suzuki, Masashi Koide, Yoshihiro Hagiwara, Eiji Itoi, Toshimi Aizawa, Masahiro Tsuchiya, Naoki Suzuki, Masashi Aoki, Makoto Kanzaki

**Affiliations:** 1 Department of Orthopaedic Surgery, Graduate School of Medicine, Tohoku University, Sendai, Japan; 2 Graduate School of Biomedical Engineering, Tohoku University, 6-6-04-110, Aramaki, Aoba-ku, Sendai, 980-8579 Japan; 3 Department of Orthopaedic Surgery, Tohoku Rosai Hospital, Sendai, Japan; 4 Department of Nursing, Tohoku Fukushi University, Sendai, Japan; 5 Department of Neurology, Tohoku University Graduate School of Medicine, Sendai, Japan

**Keywords:** Physiology, Biomarkers, Endocrinology, Biological techniques, Biological models, Cytological techniques, Cell biology, Stress signalling

## Abstract

The physiological significance of skeletal muscle as a secretory organ is now well known but we can only speculate as to the existence of as-yet-unidentified myokines, especially those upregulated in response to muscle contractile activity. We first attempted to establish an “insert-chamber based in vitro exercise model” allowing the miniature but high cell-density culture state enabling highly developed contractile human myotubes to be readily obtained by applying electric pulse stimulation (EPS). By employing this in vitro exercise model, we identified R-spondin 3 (RSPO3) as a novel contraction-inducible myokine produced by cultured human myotubes. Contraction-dependent muscular RSPO3 mRNA upregulation was confirmed in skeletal muscles of mice subjected to sciatic nerve mediated in situ contraction as well as those of mice after 2 h of running. Pharmacological in vitro experiments demonstrated a relatively high concentration of metformin (millimolar range) to suppress the contraction-inducible mRNA upregulation of human myokines including RSPO3, interleukin (IL)-6, IL-8 and CXCL1. Our data also suggest human RSPO3 to be a paracrine factor that may positively participate in the myogenesis processes of myoblasts and satellite cells. Thus, the “insert chamber-based in vitro exercise model” is a potentially valuable research tool for investigating contraction-inducible biological responses of human myotubes usually exhibiting poorer contractility development even in the setting of EPS treatment.

## Introduction

In vitro exercise models using contractile cultured myotubes, with electric pulse stimulation (EPS)^1^ application, have been widely employed for elucidating the muscle cell responses induced by actual contractile activity, including their myokine expressions^2,3^. Unlike in vivo skeletal muscles, in vitro exercise models contain only myotubes with no other cell types and thus allow solely the contraction-inducible biological responses of the myotubes to be evaluated^4,5^. This major advantage is especially valuable for investigating human myotubes since, for a variety of reasons, human exercise experiments are obviously difficult to perform as compared to animal exercise experiments. However, human myotubes derived from primary satellite cells obtained from human subjects usually exhibit much poorer contractility development than those of murine myotubes such as those originating from C2C12 myoblasts even with EPS treatment^6,7^. While we recently established a “feeder-supported in vitro exercise model” by co-culturing with murine fibroblast cells that markedly facilitated the contractility development of human myotubes^8^, a useful and simple method allowing human myotubes alone, with no escorting cells, to be readily adapted to an in vitro exercise model is eagerly anticipated.

We herein succeeded in establishing a novel “in vitro exercise model” applicable to human-derived muscle cells, which is based on utilization of a specialized insert chamber that efficiently delivers EPS to myotubes grown on a porous polyethylene terephthalate (PET) membrane. Given that our previous study indicated high-cell-density inoculation of human primary myoblasts to provide slightly better contractility development of human myotubes^7^, we used the insert, allowing miniaturization of cell culture conditions with more than ten-fold higher cell density than those in routine in vitro exercise models^7^. By employing this new “insert-chamber based in vitro exercise model” for myotubes derived from human primary satellite cells obtained from biopsy samples, we found human R-spondin 3 (RSPO3) expression to be markedly increased in myotubes in response to EPS treatment along with upregulation of other contraction-inducible factors including interleukin-6 (IL-6), IL-8, CXCL1, and striated muscle activator of Rho signaling (STARS). Moreover, our results suggest that muscular RSPO3 serves to regulate myotube development of myoblasts/satellite cells as a paracrine factor.

## Methods

### Materials

Dulbecco’s modified Eagle’s medium (DMEM) and Ham’s F-10 medium were obtained from Fujifilm Wako Pure Chemical Corp. (Osaka, Japan). Penicillin/streptomycin and trypsin–EDTA were purchased from Thermo Fischer Scientific (Rochester, NY, USA). Cell culture equipment and rectangular 8-well plates were obtained from BD Biosciences (San Jose, CA, USA) and Thermo Fisher Scientific, respectively. SUMILON Cell-disk LF1 was from Sumitomo Bakelite Co. LTD (Tokyo, Japan). Insert chamber (Falcon 353,095–353,097) was purchased from Corning (Lowell, MA, USA) and an ultrasonic cutter (ZO-91, Echo Tech Co. Ltd, Japan) was used to make an incision (~ 3 mm) for conducting electricity at the sidewall located ~ 5 mm above the bottom (Fig. S1). The insert chamber was placed between the carbon electrodes, one of which directly faced the incision. Calf serum (CS) and fetal bovine serum (FBS) were obtained from BioWest (Nuaille, France). Matrigel was obtained from Corning (#354230, NY, USA). Unless otherwise noted, all chemicals were of the purest grade available from Sigma Chemical (St Louis, MO, USA) or Fujifilm Wako Pure Chemical Corp.

### Cell culture

Primary human satellite cells were obtained by fluorescent-activated cell sorting (FACS) from muscle biopsy tissues at Tohoku University Hospital under the approval of the Ethics Committee of Tohoku University, and written informed consent was obtained from the patient (*see below*). The primary human satellite cells were amplified and stocked in liquid N_2_ as previously reported^9^. Primary satellite cells were cultured in a growth medium containing DMEM/Ham’s F10 mixture supplemented with 20% FBS, 1% penicillin–streptomycin, 1% chicken embryonic extract (United States Biological, Salem, MA, USA), and 2.5-ng/ml basic fibroblast growth factor (Thermo Fischer Scientific, Waltham, MA, USA) at 37 °C under a 5% CO2 atmosphere.

Human myoblasts (activated satellite cells) were seeded at a density of 6.6 × 10^4^ cells in the insert chamber placed on a 24-well plate in 1 mL of growth medium. We used the insert (culture area = 0.47 cm^2^, the size of which corresponds to a well of the 24-well plate) and seeded human myoblasts in a high-cell-density state (6.6 ~  × 10^4^ cells/0.47 cm^2^/insert), resulting in an approximately 12-fold higher cell density than those in routine in vitro exercise models using an 8-well culture plate (1.25 × 10^5^ cells/10.5 cm^2^/well)^7^. For immunofluorescent analysis, human myoblasts were seeded onto a Cell-Disc LF in a 24-well plate at a density of ~ 1 × 10^5^ cells. Two days after plating, differentiation was induced by switching the growth medium to DMEM supplemented with 5% horse serum, 30 µg/ml penicillin, and 100 µg/ml streptomycin (differentiation medium). The differentiation medium was changed every 24–48 h during the 7–8 days of differentiation.

In some experiments, C2C12 cells were also used for evaluating the insert-chamber-based in vitro exercise model (Fig. S1). C2C12 myoblasts were seeded at a density of 1 × 10^4^ cells in the insert chamber placed on a 24-well plate in 1 mL of DMEM supplemented with 20% FBS. Two days after seeding, differentiation was induced by switching to differentiation medium^1^ and the cells were then subjected to the EPS experiments. The differentiation medium was changed every 24–48 h during the 7–8 days of differentiation.

### siRNA-mediated reduction of RSPO3

Silencer select siRNAs against human RSPO3 (s39542, s39543), scramble siRNAs (#4,390,844, #4,390,847) and Lipofectamine RNAi MAX transfection reagent were purchased from Thermo Fischer Scientific (Grand Island, NY, USA). For myogenesis experiments, siRNAs were transfected by Lipofectamine RNAi MAX at day 0 of differentiation and then continuously cultured for 6–7 additional days with differentiation medium. For myotube experiments, siRNAs were transfected at day 5 of differentiation and cultured for 2 additional days with differentiation medium, and the cells were then used for the EPS experiments.

### Electrical pulse stimulation (EPS)

After 7–8 days of differentiation, the insert chamber and the Cell-Disc were transferred to an 8-well plate (two chambers or two Cell-Discs per well) placed in a C-Dish (IonOptix, Milton, MA) for EPS. EPS (1 Hz, 1 ~ 6 ms, 20 V/25 mm) was applied to the cells in the C-Dish using a C-Pace 100 pulse generator (IonOptix). DMEM containing 2% CS supplemented with final two-fold concentrations of amino acids, achieved by adding 50X amino acid solution (Thermo Fisher, #11130036) and 100X non-essential amino acid solution (Thermo Fisher, #11140050), was used during the EPS treatments, as previously reported^1^. A total of 4, 8, 16 or 24 h of EPS was applied and the cells were then harvested for analyses. In the experiments with a total of 16 or 24 h of EPS, 1 h intervals were set between the 8 h EPS sessions to prevent unwanted detachment of vigorously contracting myotubes from the substratum of the insert-chamber^7^.

### Western blot analysis

The cells were harvested in lysis buffer (50 mM Tris–HCl, pH7.4, 150 mM NaCl, 20 mM sodium pyrophosphate, 10 mM NaF, 2 mM sodium orthovanadate, 1 mM EDTA, 1% Triton X-100, 1 μg/ml pepstatin, 5 μg/ml leupeptin, 1 mM phenylmethylsulfonyl fluoride, 6500 IU/ml aprotinin, phosphatase inhibitor mixture-1, Sigma), and after end-over-end rotation of the homogenates for 30 min, lysate supernatants were collected by centrifugation (15,000 rpm) for 20 min at 4°C, and protein concentrations were measured using the bicinchoninic acid method with bovine serum albumin (BSA) as the standard (Pierce). After electrophoresis, western blotting was performed following standard procedures, and chemiluminescence was detected with ImageQuant LAS4000 mini (GE Healthcare). Quantification was performed with ImageQuant TL (GE Healthcare). Antibodies against RSPO3 (ATLAS Antibodies, #HPA029957), phospho-Acetyl-CoA Carboxylase (ACC) (Ser79) (Cell Signaling, #3661), phospho-AMPKα (Thr172) (Cell Signaling, #2535), and β-actin (Sigma, #A2228) were used for Western blot analysis.

### Immunofluorescence analysis

After the experimental treatments, cells on the Cell-Disc were washed with phosphate buffered saline (PBS) and fixed for 20 min with 2% paraformaldehyde in PBS containing 0.1% Triton X-100, then washed and blocked in PBS containing 5% CS and 1% BSA at room temperature. For immunofluorescence analysis, we used anti-sarcomeric α-actinin antibody (A7811, Sigma Chemical, St. Louis, MO) as the first antibody, and Alexa Fluor 488-conjugated anti-mouse IgG as the secondary antibody, (Thermo Fischer Scientific) at 1:100 and 1:1000 dilutions, respectively, in a solution of 1% BSA in PBS. In some experiments, we also used anti-RSPO3 antibody (EpiGenetek, #A60722) as the first antibody and Alexa Fluor 555-conjugated anti-rabbit IgG as the second antibody. The samples were mounted on glass slides with Vectashield (Vector Laboratories, Burlingame, CA, USA) and observed with a confocal fluorescence microscope (Fluoview FV-1000; Olympus, Tokyo, Japan) with an oil-immersion objective lens (PLANPON60xOSC2, NA 1.4, Olympus) and ASW v.1.3 software (Olympus). The fluorescence of DAPI and Alexa 488 was excited at 405 and 488 nm laser wavelengths and detected through BA430-470 and BA505-525 nm bandpass filters, respectively. The pinhole diameters are set automatically according to the selected dyeing method, and imaging was performed at 8.0–10 ms/pixel (Scan speed) for 1024 × 1024 (1:1) pixels applying a sequential scan mode with Kalman filtering. The areas of sarcomeric-α-actinin-positive fluorescent signals in each frame and in each myotube as well as numbers of nuclei in each myotube were evaluated employing the image calculator function of Fiji ImageJ software (NIH, Bethesda, MD, USA).

### Quantitative real-time PCR (qRT-PCR) analysis

Total RNA was extracted from cells employing TRI reagent (Molecular Research Center Inc., Cincinnati, OH, USA) and cDNA was then synthesized using a Transcriptor First Strand cDNA synthesis kit with oligo-dT primers (Roche, Basel, Switzerland). Next, qRT-PCR was performed with SsoAdvanced Universal SYBR Green Supermix, and detected with a Bio-Rad CFX Connect Systems (Bio-Rad Laboratories Inc., Hercules, CA, USA). The relative expression levels of the target genes were calculated using the 2-∆∆Ct method with reference genes. The primer sequences were as follows;

**Table:** 

Primers	Sequences, Forward (F), Reverse (R)
human CXCL1	(F) 5’-GCT TGC CTC AAT CCT GCA TC-3’ (R) 5’-GGT CAG TTG GAT TTG TCA CTG T-3’
human IL-6	(F) 5’-ATC TGG ATT CAA TGA GGA GAC T-3’ (R) 5’-TGT TCC TCA CTA CTC TCA AAT CTG-3’
human IL-8	(F) 5’-CAAACTTTCAGAGACAGCAGAG-3’ (R) 5’-ATCTAAGTTCTTTAGCACTCCTTGG-3’
human RSPO1	(F) 5’-ATCAAGGGGAAAAGGCAGA-3’ (R) 5’-CAGAGCTCACAGCCTTTGG-3’
human RSPO2	(F) 5’-TGTCCAACCATTGCTGAATC-3’ (R) 5’-TCCTCTTCTCCTTCGCCTTT-3’
human RSPO3	(F) 5’-CACCTTGGAAAGTGCCTTGA-3’ (R) 5’-TGACCTCACAGTGCACAATAC-3’
human RSPO4	(F) 5’-TTTGGCCCACCAGAACAC-3’ (R) 5’-CCGCAGGTCTTTCCATTG-3’
human STARS	(F) 5’-CTTGCCCTCCCAGGTAAAC-3’ (R) 5’-CACTGGGCTATATTTCTGTTGG-3’
human ribosomal protein lateral stalk subunit P0 (RPLP0)	(F) 5’-GGA AAC TCT GCA TTC TCG CT-3’ (R) 5’-GCA AGT GGG AAG GTG TAA TCC-3’
mouse RSPO3	(F) 5’- GAAGGGTTAGAAGCCAACAATC-3’ (R) 5’- AAGGATGCTGTAGTATATCTCGG-3’
mouse CXCL1	(F) 5’-GCT GGC TTC TGA CAA CAC TAT-3’ (R) 5’-CAA GCA GAA CTG AAC TAC CAT-3’
mouse glyceraldehyde-3-phosphate dehydrogenase (GAPDH)	(F) 5’- GGAGAAACCTGCCAAGTATGA -3’ (R) 5’- GCATCGAAGGTGGAAGAGT -3’

### Human satellite cell isolation and proliferation

This study was approved by the Tohoku University Hospital Institutional Review Board (approval numbers: 2014-1-703 and 2019-1-493), and written informed consent was obtained from the participant who provided the muscle sample. All experiments were performed in accordance with relevant guidelines and regulations. In this study, intact subscapularis, but not the disused supraspinatus (ruptured the supraspinatus tendon), muscles of 68-year-old male patient were subjected to further in vitro experiments^7^. Briefly, the muscle tissue was minced and digested with collagenase, filtered through a 70-µm cell strainer (BD Biosciences, Franklin Lakes, NJ, USA) and then subjected to immunostaining for FACS. Cells were incubated with an Fc receptor blocking solution and then labeled with fluorescein isothiocyanate (FITC)-conjugated anti-CD45 (clone HI30), FITC-conjugated anti-CD11b (clone ICRF444), FITC-conjugated anti-CD31 (clone WM59), phycoerythrin (PE)/Cy7-conjugated anti-CD34 (clone 581), allophycocyanin (APC)-conjugated anti-CD56 (clone MEM-188), and PE-conjugated anti-PDGFR*α* (clone 16A1). The negative set included blood markers CD11b and CD45, and endothelial markers CD31 and CD34. While CD34 is known to be expressed by the majority of mouse satellite cells^10^, human muscle-derived CD34^+^ cells are myogenic and adipogenic, whereas CD34^−^ cells are myogenic but not adipogenic^11^. We therefore used CD34 as a negative selection marker. Human satellite cells were defined as single live mononuclear CD11b^−^CD31^−^CD34^−^CD45^−^CD56^+^ cells. FACS was performed on a FACS ARIA II flow cytometer (BD Biosciences). Cells were cultured in a growth medium and cultured at 37 °C in a 5% CO_2_ atmosphere. When cells reached 60%-80% confluence, adherent cells were split onto a new Matrigel-coated 15-cm dish to expand the activated satellite cells. Activated satellite cells (myoblasts) were suspended in Cell Banker (TAKARA, CB011, Japan) and stored in liquid nitrogen.

### In Situ Muscle Contraction and Running Exercise

Male 7–9-week-old Balb/c mice were purchased from CLEA-Japan (Tokyo, Japan). The experimental design, as well as the care and use of the mice, followed the guidelines for animal experiments of Tohoku University and the Ethics Committee for Animal Experiments, Tohoku University, approved these studies (Ethics approval #2020BeLMO-006–01). The study was carried out in compliance with the ARRIVE guidelines and the aforementioned guidelines. The mice were kept in standard cages in an air-conditioned room under a 12:12 light/dark cycle at 23 ± 1°C. They were allowed access to standard food pellets (24.2% protein, 4.5% lipids, 56.8% carbohydrates) (Labo MR Standard, Nihon Nosan Kogyo Co., Yokohama, Japan) and tap water ad libitum. Mice that had been fasted for 16 h were anesthetized with an intraperitoneal injection of medetomidine (ZENOAQ, 0.3 mg/kg), midazolam (SANDZ, 4.0 mg/kg), and butorphanol (Meiji Seika Pharm CO, 5.0 mg/kg). The sciatic nerves were bilaterally isolated, and subminiature electrodes were placed around each nerve and interfaced with the electrical stimulation instrument (STG4004, Multi Channel Systems, Reutlingen, Germany) as previously reported^12^. Hindlimb muscles on one side were stimulated to induce contractions with 1 s on/1 s off cycles for 3 min (2.5 mA amplitude, 50 Hz, 1 ms duration), and other leg served as a sham-operated control. At 1 h after EPS, SOL and EDL muscles were isolated from the mice and then subjected to RT-PCR analysis. Our EPS setting was determined by monitoring upregulations of CXCL1 and IL-6 mRNAs in SOL and EDL muscles (Fig. S3), confirming that these two factors are well-established as exercise-inducible myokines in both in vivo^1,5^ and in vitro^4,7,13^ experimental systems.

Forced wheel running was imposed on mice at room temperature (23 ± 1 °C) for the indicated time in each experiment, as previously described but with slight modifications^14^. Briefly, each mouse was placed in a cylindrical cage (17 cm diameter, 7 cm width) with stainless steel rods (2 mm diameter, 1 cm apart), which was rotated by an electric motor at a walking speed of ~ 12 m/min. In total, 12 running wheels were operating simultaneously. The SOL and EDL muscles were isolated from the mice immediately after 2 h of running and then subjected to RT-PCR analysis.

### Statistical analysis

Statistical analyses were performed using Student’s *t*-test or ANOVA with Tukey’s multiple comparison test, and *P* values < 0.05 were considered to indicate a statistically significant difference. Data are expressed as means ± SE unless otherwise specified.

### Ethics declarations

The experimental design, care, and use of the mice were performed according to the guidelines for animal experiments at Tohoku University. Ethical approval for this study was obtained from the Animal Research Committee of Tohoku University (approval number; #2020BeLMO-006-01).

## Results

### Effect of EPS on RSPO3 mRNA expression in human myotubes cultured in the insert chamber

**Figure 1:**
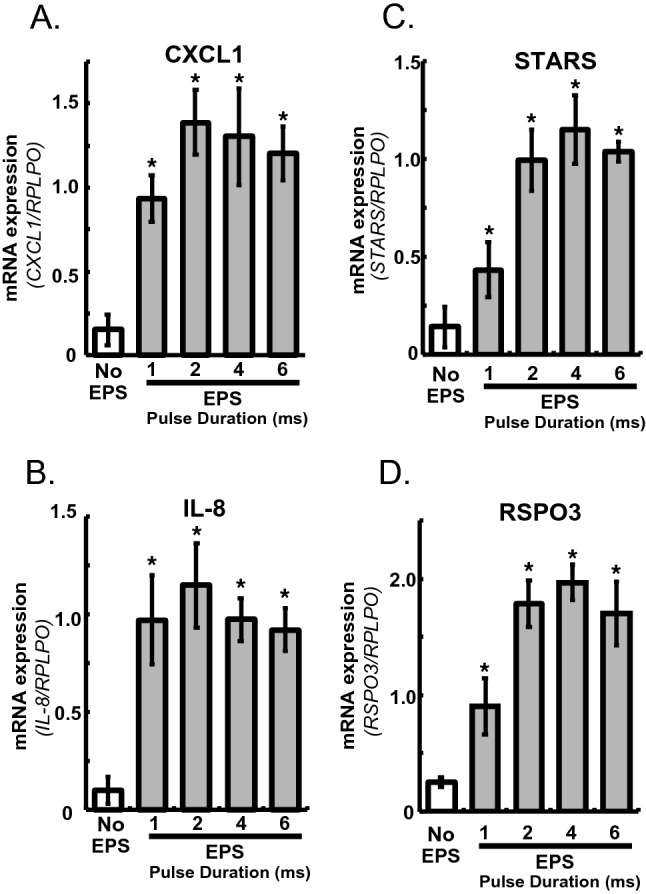
Human R-spondin 3 (RSPO3) mRNA expression evoked by EPS treatment. After 7–8 days of differentiation, the insert chambers were placed in an 8-well plate set on a C-Pace, and EPS (1 Hz, 20 V/25 mm) with a 1-, 2-, 4-, or 6-ms pulse duration was applied to the differentiated human myotubes for a total of 24 h, as described in the Methods. Total RNA samples were prepared and subjected to RT-PCR analysis to evaluate mRNA levels for CXCL1 (A), IL-8 (B), striated muscle activator of Rho signaling (STARS) (C), and RSPO3 (D). Data normalized using RPLP0 transcript were averaged over 3 independent experiments (*P < 0.05).

Given that a high-cell-density inoculation of human-origin myoblasts (activated satellite cells) improves their contractility development along with better upregulation of myokines in response to EPS^7^, we used the insert chamber allowing us to maintain the culture in a high-cell-density state (6.6 ~  × 10^4^ cells/culture), resulting in better contraction-inducible biological responses of human myotubes with EPS treatment. As shown in Figs. 1A–C, we confirmed that human myotubes in the insert chamber were highly responsive to EPS treatment as assessed by upregulations of the expressions of contraction-inducible mRNAs including those of myokines (CXCL1 and IL8)^1,7^ and STARS^15^. Since EPS (1 Hz, 20 V/25 mm) with a 2 ~ 4 ms pulse duration produced maximum responses of all mRNA upregulations examined (Figs. 1 and S1), we hereafter applied EPS with a 4 ms pulse duration for all experiments.

Preliminary RNA-seq analysis identified a significant increase in RSPO3 mRNA expression in response to EPS-induced contractile activity when using the in vitro exercise model (DDBJ repository, Accession number: PRJDB12901)*.* We performed RT-PCR analysis for RSPO1-4 mRNA expression levels and confirmed the EPS-induced RSPO3 upregulation in human myotubes cultured in the insert chamber to occur in a manner dependent on the pulse duration of EPS (Fig. 1D). In contrast, no expressions of RSPO1 and RSPO4 mRNAs were detected, and the RSPO2 mRNA expression level did not change in response to EPS in human myotubes (Fig. S2).

**Figure 2:**
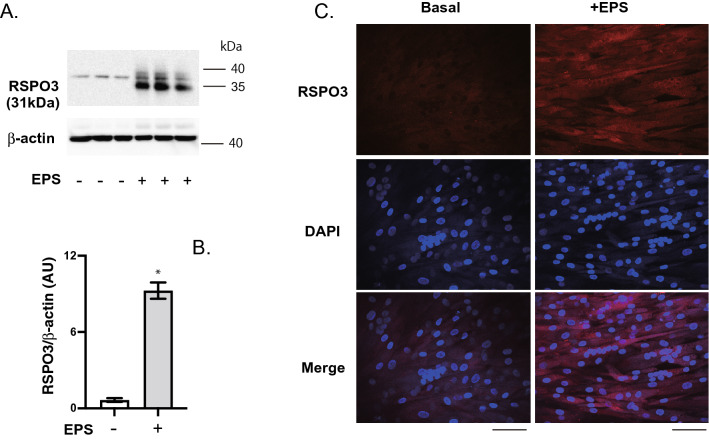
Western blotting and immunofluorescent staining analyses of human R-spondin 3 (RSPO3) protein in human myotubes. (**A**) Whole cell lysates of the differentiated human myotubes (day8) with or without 24 h of EPS in total (1 Hz, 4-ms, 20 V/25 mm) were subjected to Western blot analysis using anti-RSPO3 and anti-β-actin antibodies. Three independent experiments were performed; representative results are shown. (**B**) Results from (**A**), RSPO3 protein amounts in response to EPS, were subjected to densitometric analysis of all bands broadly detected around 35 ~ 40 kDa for quantification (*n* = 3). Data normalized using β-actin protein amounts. (**C**) Differentiated human myotubes on the Cell-Discs with and without 24 h in total of EPS treatment were fixed with paraformaldehyde-PBS and then subjected to immunofluorescent staining using anti-RSPO3 antibody (*red*). DAPI was used for nuclear staining (*blue*). Scale bar = 100 µm. Three independent experiments were performed, and representative images are presented.

Western blotting analysis of whole cell lysates from human myotubes demonstrated that EPS significantly increased the RSPO3 protein amounts broadly observed around 35 ~ 40 kDa (Fig. 2A), due perhaps to its *N*-glycosylation^16^ and *C*-mannosylation^17^. We observed approximately 37 kDa bands even in the control myotubes, but their nature and relationships to other EPS-inducible bands broadly detected around 35 ~ 40 kDa are unclear. Densitometric evaluation of all bands, including that at 37 kDa, demonstrated significant increases in RSPO3 protein amounts after EPS treatment (Fig. 2B). The EPS-dependent increase in RSPO3 protein was further confirmed by immunofluorescent staining analysis using anti-RSPO3 antibody (Fig. 2C).

### Characterization of RSPO3 expression in human cultured muscle cells

**Figure 3:**
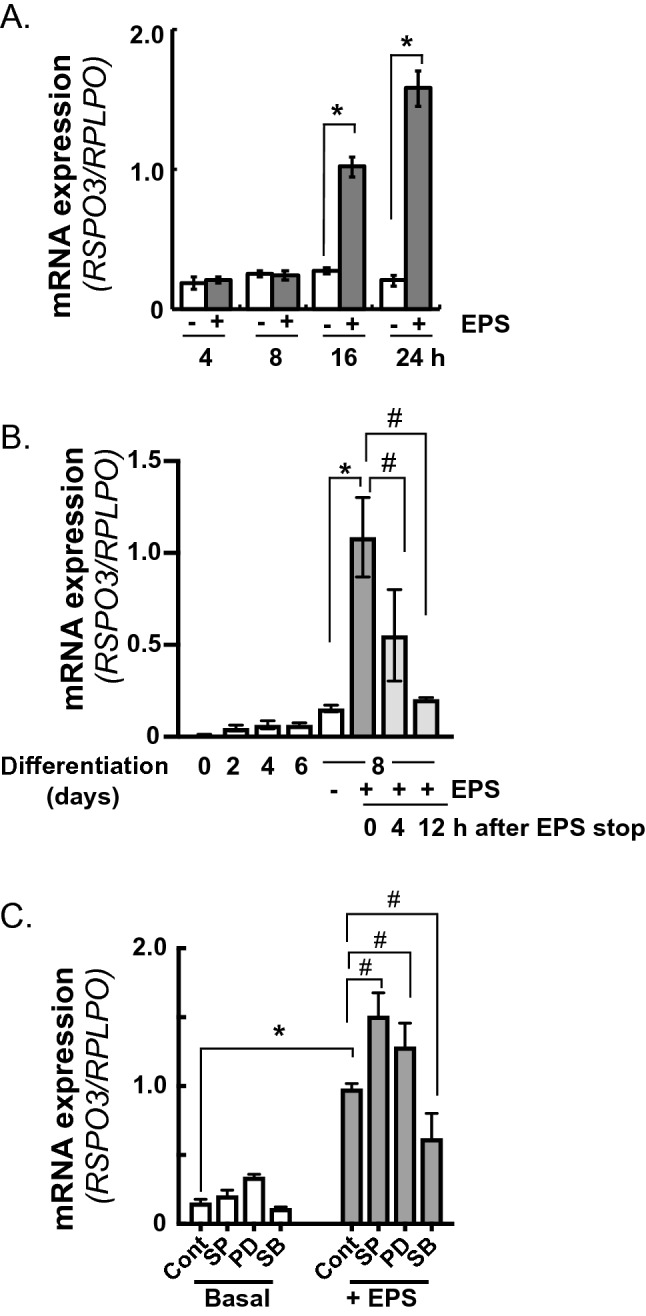
Human R-spondin 3 (RSPO3) mRNA expression during myogenesis and effects of MAPK inhibitors on EPS-induced RSPO3 upregulation. (**A**) Time-course of RSPO3 mRNA expression of differentiated human myotubes (day8) in response to the indicated duration of EPS (1 Hz, 4-ms, 20 V/25 mm) were evaluated by RT-PCR. Data normalized using RPLP0 transcript were averaged over 3 independent experiments (*P < 0.05). (**B**) RSPO3 mRNA levels during myogenesis on the indicated day and those of fully differentiated human myotubes (day 8) subjected to EPS (1 Hz, 4-ms, 20 V/25 mm) were evaluated by RT-PCR. Effects of EPS cessation on RSPO3 mRNA expression at the indicated time were also evaluated in fully differentiated human myotubes (day 8) after 24 h of EPS treatment. (**C**) EPS was applied in the presence or absence of SP-600129 (SP; 20 µM), PD-98056 (PD: 50 µM), or SB-203580 (SB; 5 µM), and RSPO3 mRNA expression levels were evaluated by RT-PCR. Data normalized using RPLP0 transcript were averaged over 3 independent experiments. Multi factor ANOVA with Tukey’s multiple comparison test was used for statistical analyses; the significance of the effect of EPS is denoted as *P < 0.05; the effects of time after EPS cessation vs. immediately (0 h) after EPS (B), or the effects of reagents compared to the control (with EPS) (C) are denoted as # < 0.05.

To examine RSPO3 expression during differentiation of human myoblasts, we performed RT-PCR analysis and found that RSPO3 expression was slightly increased upon myogenic differentiation. In fully differentiated myotubes (day 8), RSPO3 expression was markedly upregulated by EPS treatment (Fig. 3A). Contraction-inducible RSPO3 upregulation diminished to ~ 50% at 4 h after stopping EPS and returned to the basal level the next day, indicating that RSPO3 mRNA upregulation is entirely dependent on contractile activity elicited by EPS treatment in differentiated myotubes under the culture conditions employed in this study (Fig. 3B).

Given that several MAP-kinase signaling cascades appear to be involved in EPS-dependent myokine expressions in myotubes^13^, pharmacological inhibitors were used to examine whether EPS-dependent RSPO3 mRNA upregulation was impacted by chemical reagents (Fig. 3C). EPS-induced RSPO3 upregulation was enhanced in the presence of SP-600129 (JNK inhibitor), or PD-98056 (MEK1/2 inhibitor), whereas SB-203580 (p38 MPAK inhibitor) slightly but significantly inhibited RSPO3 upregulation (Fig. 3C), implying that these MAP kinase cascades play intricate roles in the observed contraction responsiveness.

### Effects of siRNA-mediated silencing of RSPO3 on EPS-inducible myokine upregulation and myogenic differentiation

**Figure 4:**
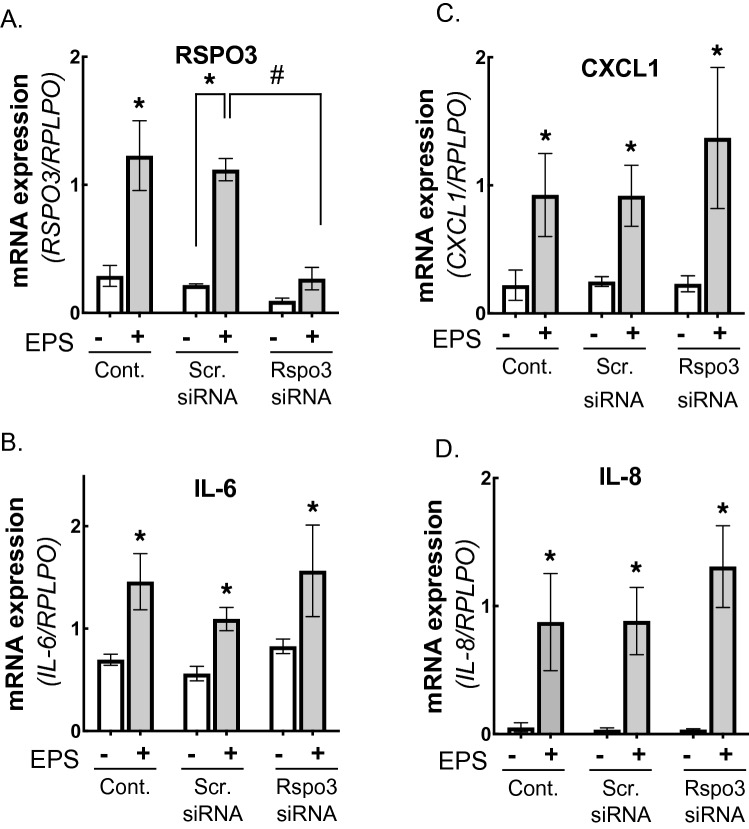
Effects of siRNA-mediated R-spondin 3 (RSPO3) silencing on EPS-induced expressions of other myokines in human myotubes. EPS (1 Hz, 4-ms, 20 V/25 mm) was applied to the differentiated human myotubes treated with siRNAs as described in the Methods. Total RNA samples were prepared and subjected to RT-PCR analysis to evaluate mRNA levels for RSPO3 (**A**), IL-6 (**B**), CXCL1 (**C**) and IL-8 (**D**). Data normalized using RPLP0 transcript were averaged over 3 independent experiments. Multi factor ANOVA with Tukey’s multiple comparison test was used for statistical analyses; the significance of the effect of EPS is denoted as *P < 0.05; the effect of sRNAs compared to the scramble siRNAs control is denoted as # < 0.05. Note that RSPO3 expression is effectively reduced by RSPO3 siRNAs but not by scramble control siRNAs.

To examine the possible involvement of RSPO3 in the processes by which other myokines are upregulated in response to EPS-evoked contraction, siRNA-mediated RSPO3 knockdown experiments were carried out. In this set of experiments, siRNAs were transfected into myotubes 2 days before EPS treatment. Under conditions wherein RSPO3 expression was significantly suppressed by specific, but not scramble, siRNA pretreatment, no obvious impact of RSPO3 silencing was detected and EPS-dependent upregulations of IL-6, IL-8 and CXCL1 were similar to those observed in the control groups (Fig. 4). These results suggest that RSPO3 is not directly involved in EPS-inducible upregulation of several other myokines, at least not IL-6, IL-8 and CXCL1, in differentiated myotubes.

**Figure 5:**
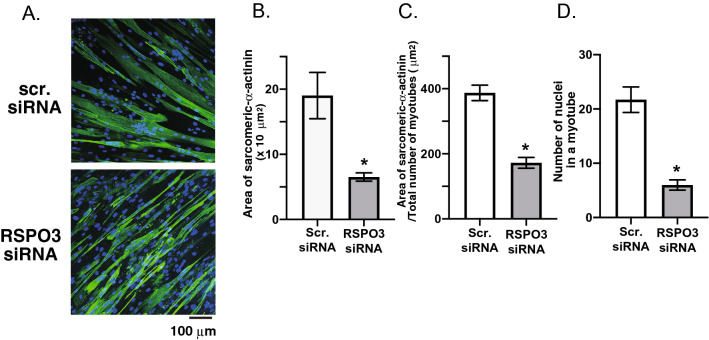
Effect of siRNA-mediated R-spondin 3 (RSPO3) silencing on human myotube formation. (**A**) The siRNAs against RSPO3 or control scramble RNAs were transfected twice into human myoblasts on day 0 and on day 2, and then cultured further for total 7–8 days in differentiation medium. Fixed cells were subjected to immunofluorescent staining using anti-sarcomeric-α-actinin antibody (*green*). Three independent experiments were performed and representative images are presented. The total area of sarcomeric-α-actinin-positive fluorescent signals per field of view (**B**), area of sarcomeric-α-actinin-positive fluorescent signals in a human myotube (**C**), and numbers of nuclei in a myotube (**D**) were evaluated as described in the Methods. Summarized graphs averaged over 3 independent experiments are shown on the right (*P < 0.05).

The involvement of RSPOs, particularly RSPO1 and 2, in myogenic differentiation and fusion processes has previously been reported^18,19^. We therefore examined effects of siRNA-mediated silencing of RSPO3 in myoblasts on myotubular formation (Fig. 5A) as assessed by sarcomeric α-actinin immunofluorescent staining analysis and found that RSPO3 knockdown significantly compromised myogenic differentiation in terms of both the areas of myotubes formed (Fig. 5B and C) and the numbers of nuclei in a myotube (Fig. 5D).

### Effect of metformin on EPS-dependent RSPO3 upregulation in human myotubes

**Figure 6:**
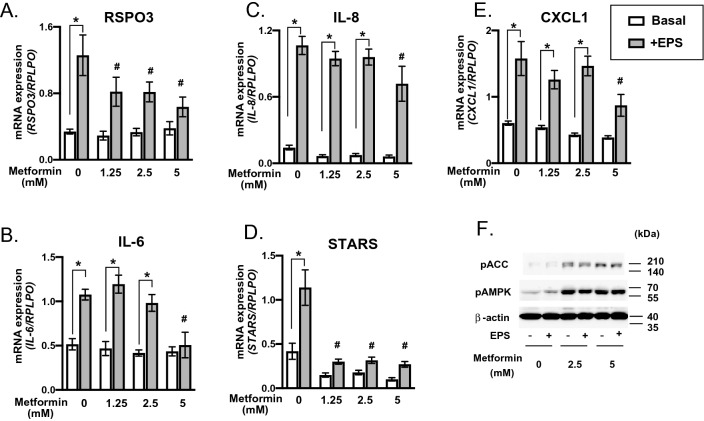
Effects of metformin on EPS-inducible upregulation of human R-spondin 3 (RSPO3) and other factors. EPS (1 Hz, 4-ms, 20 V/25 mm) was applied to the differentiated myotubes in the presence or absence of the indicated concentrations of metformin. Total RNA samples were prepared and subjected to RT-PCR analysis to evaluate mRNA levels for RSPO3 (**A**), IL-6 (**B**), IL-8 (**C**), CXCL1(**D**), and striated muscle activator of Rho signaling (STARS) (**E**). Data normalized using RPLP0 transcript were averaged over 3 independent experiments. Multi factor ANOVA with Tukey’s multiple comparison test was used for statistical analyses; the effect of EPS is denoted as *P < 0.05; the effect of metformin is denoted as # < 0.05. (**F**) Whole cell lysates of the differentiated human myotubes (day8) with and without a total of 24 h of EPS (1 Hz, 4-ms, 20 V/25 mm) in the presence of metformin (0, 2.5, 5 mM) were subjected to Western blot analysis using anti-phospho-acetyl-CoA carboxylase (pACC), anti-phospho-AMP-activated kinase (pAMPK), and anti-β-actin antibodies. Three independent experiments were performed; representative results are shown.

Despite the therapeutic actions of metformin^20^, several lines of evidence indicate that metformin partially blunts some of the beneficial adaptations elicited by exercise training^21,22^ as well as muscle performance^23^. We therefore examined the effect of metformin on contraction-inducible gene upregulations including that of RSPO3 and found that a relatively high concentration of metformin (~ 5 mM) significantly suppressed EPS-dependent upregulations of RSPO3, IL-6, IL-8 and CXCL1 mRNA expressions (Figs. 6A–E). The upregulations of the mRNAs of RSPO3 (Figs. 6A) and STARS (Figs. 6D) were found to be more sensitive to metformin and, in fact, a lower concentration (1.25 mM) of metformin significantly inhibited the EPS-dependent response. Western blot analysis confirmed that metformin markedly stimulated phosphorylation of both AMPK and ACC (Fig. 6F). These results suggest EPS-dependent mRNA expression of several myokines including RSPO3 to be negatively affected by the presence of metformin.

### Effects of sciatic-nerve-mediated muscle contraction and running exercise on RSPO3 expression in soleus and EDL muscles

**Figure 7:**
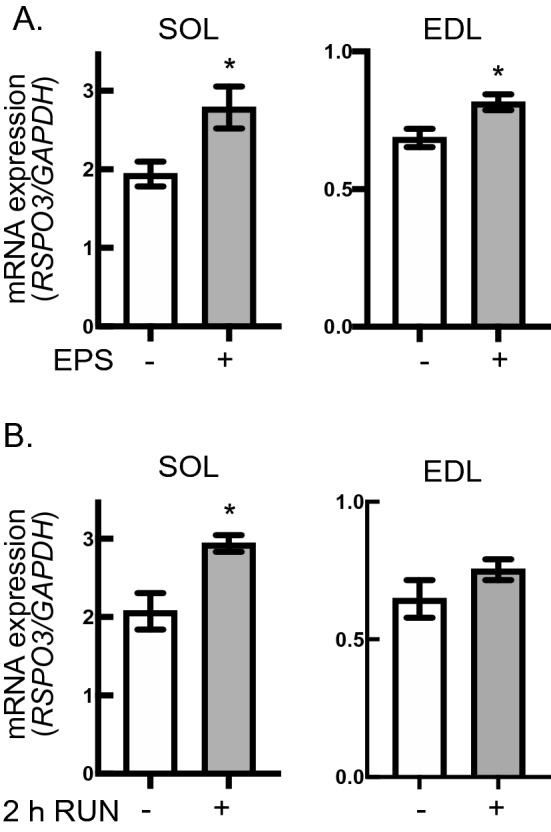
Mouse R-spondin 3 (RSPO3) upregulation upon contraction of in vivo skeletal muscles from mice. (**A**) After the in situ muscle contraction via sciatic nerve-mediated EPS treatment as described in the Methods, soleus (SOL) and extensor digitorum longus (EDL) muscles were obtained and subjected to RT-PCR analysis to evaluate mRNA levels for RSPO3. Data were normalized using mouse GAPDH transcripts and compared to those of the muscle tissues obtained from sham-operated control (opposite side) legs (*n* = 9; *P < 0.05). (**B**) After 2 h of running (a running speed of ~ 12 m/min), SOL and EDL muscles were obtained and subjected to RT-PCR analysis. Data were normalized using mouse GAPDH transcripts and compared to those of the muscle tissues obtained from sedentary control mice (*n* = 5 each; *P < 0.05).

To examine contraction-dependent muscular RSPO3 upregulation in vivo, we utilized an in situ mouse muscle contraction model with sciatic nerved-mediated electrical pulse stimulation (SN-EPS). RSPO3 mRNA levels increased significantly in both EDL and SOL muscles in response to SN-EPS treatment as compared with those in sham control legs (Fig. 7A). As positive controls, increased expressions of IL-6 and CXCL1 mRNAs were confirmed in EDL and SOL muscles which had undergone SN-EPS-mediated contractile activity (Figure S3).

Wheel running experiments (2 h) demonstrated RSPO3 mRNA levels to be significantly increased in SOL muscles. Although EDL muscle showed a tendency for RSPO3 mRNA upregulation in response to 2 h of running, the increase did not reach statistical significance (Fig. 7B).

## Discussion

While “in vitro exercise models”, employing mostly murine muscle cells such as C2C12 cells, have been widely used for investigating contraction-inducible biological responses including myokine expressions^1,13,24^, human-derived myotubes have generally exhibited much poorer contractility development, resulting in minimal myokine expressions even with EPS treatments^6,7^. In the present study, we established an “insert chamber-based in vitro exercise model” applicable to human myotubes that allows highly-developed EPS-responsive human myotubes to be readily obtained. By using the insert possessing electric conductive path on the wall, myotubes on the PET membrane in the insert could be effectively exposed to EPS and thus exhibited myokine upregulations sensitively in a manner dependent on EPS intensity (e.g., pulse duration) (Fig. S1). EPS-inducible biological responses in the insert chambers exhibited an EPS-intensity dependence, being maximal at a 2 ~ 4 ms EPS pulse duration, essentially the same responsiveness as that observed in the conventional culture plate^1,4,7,8^. Therefore, miniaturization along with higher-cell-density using the insert not only contributes to better development of human myotube contractility but also allows for marked increases in sample numbers, which would be an important advantage especially for primary human myotubes derived from biopsy samples, a very limited resource.

By employing our “insert chamber-based in vitro exercise model comprised of primary human myotubes”, we focused on the effects of EPS on human RSPO3 mRNA expression since exercise-dependent regulation of RSPO3 expression had yet to be investigated, despite its physiological significance in various muscular functions having been revealed in experimental animal studies^18,19,25–29^.The findings presented herein demonstrate that mRNA expression of RSPO3, but not other RSOPs, in human myotubes is highly sensitive to EPS treatment (Figs. 1 and S2), features very similar to those of other established contraction-inducible myokines including IL-6, IL-8, and CXCL1^1,4,13^. Western blotting and immunofluorescent staining analyses confirmed that human myotubes did indeed produce RSPO3 protein in response to EPS treatment (Fig. 2). While these results clearly indicated RSPO3 to be produced by human myotubes especially in response to EPS treatment, we were not able to measure RSPO3 proteins secreted into the culture medium by the ELISA method, due to detection limits of the assay. We used a small insert (culture area = 0.47 cm^2^) to optimize cell density and then placed two of these inserts in a well of an 8-well plate containing a relatively large volume of culture medium (5 mL/10.5 cm^2^/well). Therefore, we can reasonably assume that a small amount of RSPO3 secreted into the medium would be extremely diluted and thus be present at a concentration below the detection limit of the assay.

Interestingly, as we reported previously for EPS-dependent IL-6 upregulation in murine C2C12 myotubes^4^, EPS-induced RSPO3 mRNA upregulation was relatively delayed and a significant increment was observed after 16 h of EPS treatment (Fig. 3A). Importantly, EPS-induced RSPO3 mRNA upregulation was restored to the basal level after cessation of EPS (Fig. 3B), further illustrating RSPO3 expression to be predominantly regulated by the EPS-dependent contractile activity in myotubes, possibly serving as an exercise factor in vivo. Consistent with this notion, we observed RSPO3 mRNA in mouse skeletal muscles to be significantly upregulated in both SOL and EDL muscles, subjected to SN-EPS-evoked in situ contraction, while no such elevation was detected in those of sham control legs (Fig. 7). Exercise-inducible upregulation of muscular RSPO3 was further confirmed in the SOL muscles after 2 h of running, while EDL muscles did not exhibit significant RSPO3 upregulation (Fig. 7B). Although it is quite difficult to directly compare these in vitro results with data obtained in vivo, one of the explanations for the discrepancy in the time differences required for RSPO3 expression between in vitro and in vivo is that acquisition of RSPO3 upregulation capability in myotubes may represent the maturation process of contraction-dependent responsiveness, as is similarly observed with IL-6 that reportedly required more than 12 h of EPS^4^. Taking these in vitro and in vivo data together, our findings provide compelling evidence that RSPO3 expression is regulated by muscle contractile activity and also suggest that RSPO3 serves as a contraction-inducible myokine in vivo.

RSPOs are secretory proteins that play important roles in cell proliferation, differentiation and death by evoking the Wnt signaling pathways in various cell types^30^. In the skeletal muscle research field, physiological roles of RSPOs, especially RSPO1-3, have been well documented in myogenesis^18,25^, muscle development^26^, muscle repair/regeneration^19,27^, and acetylcholine receptor clustering^28,29^ in animal models including mice and xenopus, though RSPO studies in human muscle cells have been lacking. Thus, based on our present findings, future human studies are warranted to determine whether and how muscular RSPO3 expression is stimulated by physical activity and/or contractile activity of skeletal muscles in vivo.

Pharmacological and siRNA-mediated silencing experiments conducted in the present study provide important insights into regulatory mechanism(s) involving contraction-dependent RSPO3 mRNA upregulation as well as into its possible physiological roles in muscle cells. As is evident from Fig. 3C, multiple MAP kinase cascades play intricate roles in RSPO3 expression in both positive and negative fashions in human myotubes. Unlike the contraction-dependent CXCL1 upregulation that reportedly relies mainly on the JNK MAPK pathway^13^, SP600129, a JNK inhibitor, slightly but significantly augmented RSPO3 upregulation, a phenomenon which was also detected in contraction-induced IL-6 upregulation^4^. PD98056, a MEK1/2 inhibitor, also exerted a positive effect on RSPO3 upregulation, while SB20358, a p38 inhibitor, slightly but significantly suppressed this response. Thus, intracellular signaling pathways involved in the contraction-dependent upregulation of each myokine apparently differ from each other, and the EPS-induced RSPO3 upregulation is likely to be balanced by the multiple MAP kinase cascades that may contribute to deciphering the complicated signals evoked by the actual contractile activity of myotubes. Clarifying intracellular signaling networks involved in the contraction-dependent myokine upregulations is fundamental for our understanding of exercise physiology^31^, and this important issue needs to be addressed by future experiments.

An interesting observation made in the pharmacological experiments depicted in Fig. 6 was that a relatively high concentration of metformin (~5 mM), the first-line drug for type 2 diabetes^32,33^, slightly but significantly suppressed the upregulation of all contraction-inducible myokines examined in the present study including RSPO3 (Fig. 6). Intriguingly, EPS-induced expressions of RSPO3 and STARS mRNAs were moderately more sensitive to this reagent and a lower concentration of metformin (1.25 mM) significantly suppressed their upregulations in response to EPS treatment. However, the millimolar range of metformin doses, representing supra-pharmacological concentrations, was required for the inhibitory effect on contraction-inducible responses including those of RSPO3 and STARS, suggesting mitochondrial respiratory chain complex I inhibition and thereby AMPK activation^34,35^ to likely be involved in the suppressive effects. Indeed, AMPK phosphorylation was markedly augmented by metformin (2.5 mM) treatment in human myotubes (Fig. 6). Given that metformin reportedly dampened some beneficial adaptations induced by exercise training in several clinical trials^21,22,36^, our in vitro results suggest that the impact of metformin on the contraction-inducible expressions of myokines including RSPO3 may, by as yet unknown mechanisms, be related to the clinical effects of metformin on exercise benefits. Although we did not observe obvious cytotoxic effects of metformin as assessed by myotube morphology in the present study, a high concentration of metformin reportedly influenced cellular viability^37^ and therefore caution must be exercised when interpreting the in vitro data particularly for supra-physiological concentrations of metformin. In any case, our “insert chamber-based in vitro exercise model” for human myotubes is a potentially valuable research tool for directly examining pharmacological actions on contracting human myotubes in greater detail, and future studies employing more sophisticated experimental conditions are necessary to understand the relationship between muscle functions, e.g., contractility, and metformin actions.

As mentioned above, the physiological roles of RSPOs, including RSPO3, in muscle functions have been intensively studied mostly by using experimental animals^18,19,25–29^ and their positive myogenic actions on myoblasts and satellite cells are well established. For example, RSPO2 reportedly enhanced the expression of MYF5, a key myogenic determination factor, mediated through WNT/β-catenin signaling, leading to hypertrophic myotube formation, and siRNA-mediated silencing of either RSPO2 or RSPO3 significantly compromised MYF5 expression in murine C2C12 myoblasts^18^. Consistent with the evidence obtained using animal models, we confirmed that siRNA-mediated RSPO3 silencing in human myoblasts inhibits their myotube formation (Fig. 5), implying the significance of RSPO3 for human muscular functions. Thus, our present findings not only confirmed the physiological significance of RSPO3 serving as a myogenic stimulator in human muscle cells but also strongly suggest that exercise-inducible RSPO3 serves as a local intramuscular factor which regulates myogenic cells neighboring the working myofibers that might be involved in hypertrophy and/or repair processes after exercise. Future studies are warranted to directly test whether RSPO3 functions as a myokine in regulating muscle functions by modulating myoblasts/satellite cells in vivo.

Given that siRNA-mediated silencing of RSPO3 in myotubes failed to exert any obvious effect on EPS-dependent upregulation of other myokines including IL-6, IL-8 and CXCL1 (Fig. 4), augmented RSPO3 expression is highly unlikely to be involved directly in the regulation of expressions of these myokines, at least as an autocrine factor. Taking these data together with those obtained in previous studies, the enhanced RSPO3 expression in response to contractile activity may serve as a positive myogenic factor for neighboring myoblasts/satellite cells, acting in a paracrine manner in contracting muscle tissues in vivo. Moreover, RSPO3 reportedly influenced, in animal studies, the body fat distribution via regulation of adipogenesis^38^, and hepatic cholesterol synthesis^39^. Thus, muscle derived RSPO3 enhanced by contractile activity might have an impact on these phenomena in adipose and/or liver tissues as an endocrine factor. In any case, further experiments are needed to define the physiological roles of muscle RSPO3 that is markedly enhanced by muscle cell contractile activity.

In summary, we herein provide compelling evidence that RSPO3 mRNA expression is highly upregulated in human myotubes by EPS treatment. These findings were obtained using a newly established “in vitro exercise model”, which is based on the utilization of a specialized insert chamber. Our approach, using the insert chamber, further expands the usefulness of the “in vitro exercise model”, allowing us to maximize the use of human muscle cells obtained from clinical biopsy samples, a very limited resource.

## Supplementary Information

Supplementary Information 1.

Supplementary Information 2.

## Data Availability

The RNA-seq datasets generated during the current study are available in the DDBJ repository, Accession No.: PRJDB12901. https://ddbj.nig.ac.jp/resource/bioproject/PRJDB12901. The data that support the findings of this study are available from the corresponding author, MK, upon reasonable request.
