# Supplementary material for: RSPO3 is a novel contraction-inducible factor identified in an “in vitro exercise model” using primary human myotubes
Source: Sci Rep. 2022 Aug 22;12:14291. doi: 10.1038/s41598-022-18190-z (PMC9395423; doi:10.1038/s41598-022-18190-z)

Supplementary Information:

RSPO3 is a novel contraction-inducible factor identified in an "in vitro exercise model" using primary human myotubes

Tadahisa Takahashi, Yuqing Li, Weijian Chen, Mazvita R. Nyasha, Kazumi Ogawa, Kazuaki Suzuki, Masashi Koide, Yoshihiro Hagiwara, Eiji Itoi, Toshimi Aizawa, Masahiro Tsuchiya, Naoki Suzuki, Masashi Aoki and Makoto Kanzaki

Original blot
(Figure 2)

anti-RSPO3
antibody

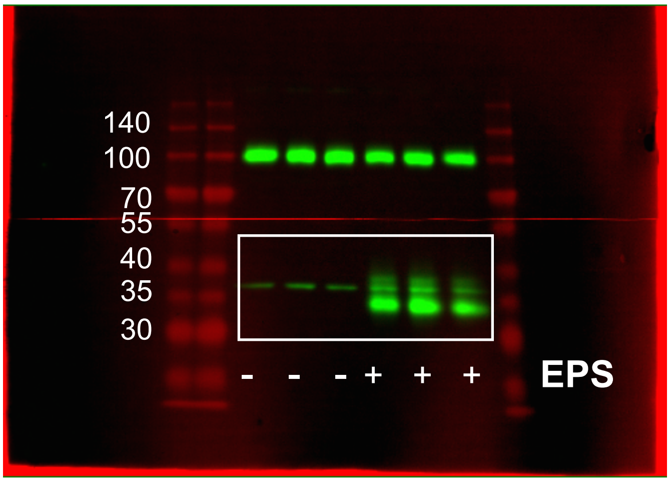

anti-β-actin
antibody

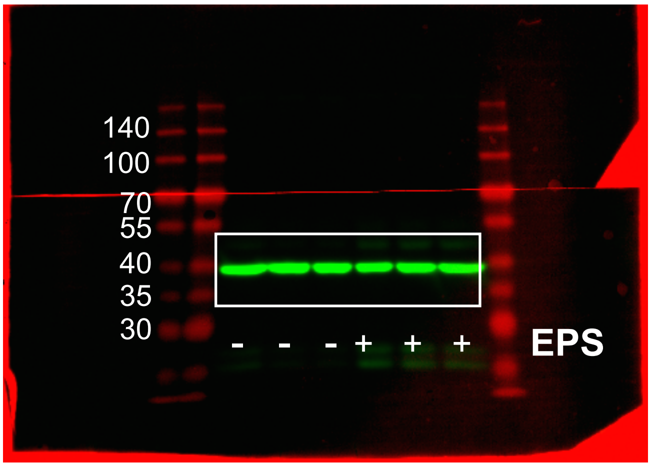

Original blot
(Figure 6)

anti-pACC (Ser79)
antibody

anti-pAMPK(Ser172)
antibody

anti-β-actin
antibody

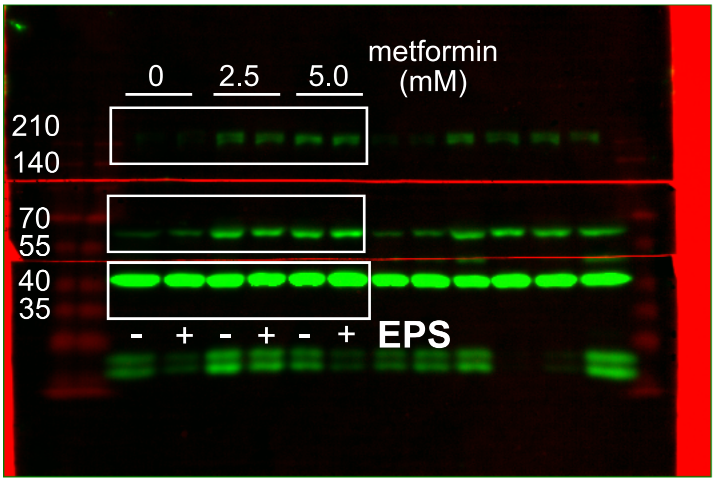

Supplement: Supplementary file 1 — Supplementary Information 1. [file 41598_2022_18190_MOESM1_ESM.pdf]
