# Supplementary material for: RSPO3 is a novel contraction-inducible factor identified in an “in vitro exercise model” using primary human myotubes
Source: Sci Rep. 2022 Aug 22;12:14291. doi: 10.1038/s41598-022-18190-z (PMC9395423; doi:10.1038/s41598-022-18190-z)
Supplement: Supplementary file 2 — Supplementary Information 2. [file 41598_2022_18190_MOESM2_ESM.pdf]

Supplementary Figures:

RSPO3 is a novel contraction-inducible factor identified in an "in vitro exercise model" using primary human myotubes

Tadahisa Takahashi, Yuqing Li, Weijian Chen, Mazvita R. Nyasha, Kazumi Ogawa, Kazuaki Suzuki, Masashi Koide, Yoshihiro Hagiwara, Eiji Itoi, Toshimi Aizawa, Masahiro Tsuchiya, Naoki Suzuki, Masashi Aoki and Makoto Kanzaki

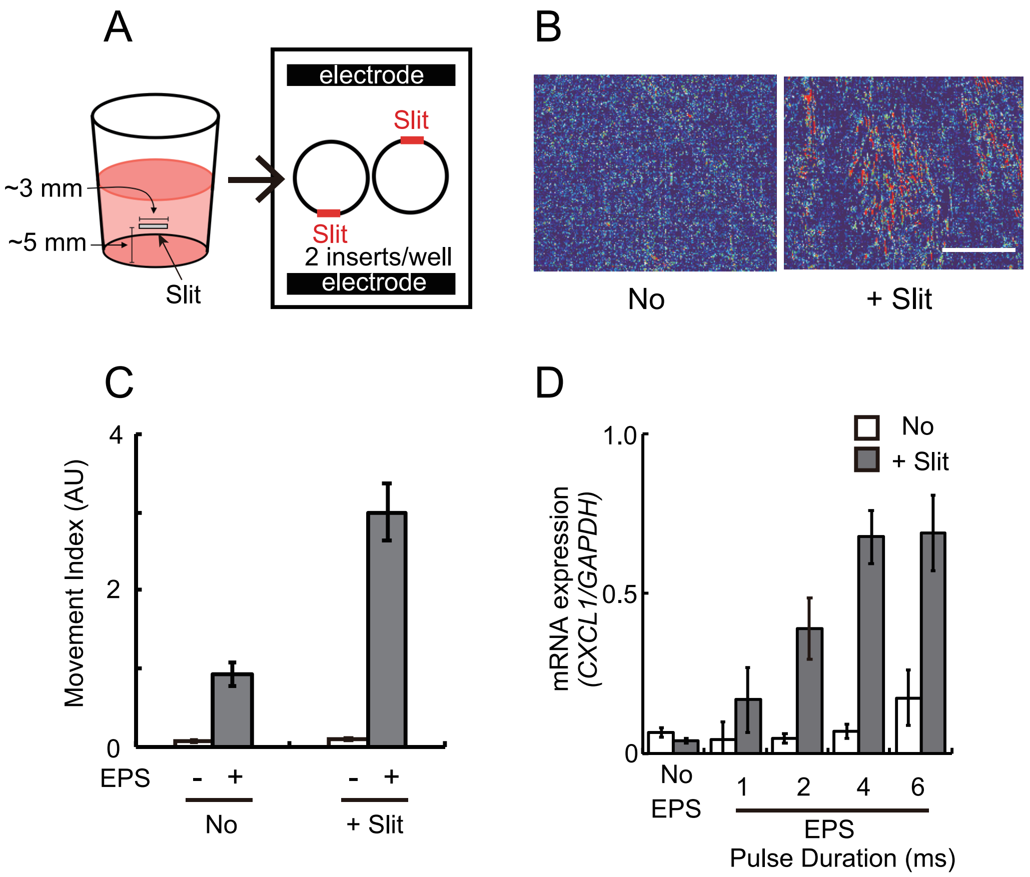

Figure S1. Schematic diagram of insert chamber setting (A) and effects of EPS on contractility (B, C) and CXCL1 mRNA expression (D) in C2C12 myotubes

(A) C2C12 myoblasts were seeded in an insert chamber possessing either a slit or no-slit. After 7-8 days of differentiation, the insert chamber was transferred to an 8-well plate placed in a C-Dish for EPS. Two inserts were placed between the carbon electrodes, one of which directly faced to the slit, in a well (5 mL medium) of an 8-well plate. For evaluating the EPS-evoked contractile activity, the movies were taken at the end (the last ~10 min) of a total 24-h EPS (1Hz, 4-ms, 20V/25 mm) treatment period. (B, C) The index of movement was calculated by the differential image subtraction method. The pseudo-colored differential images, reflecting contractile area and ability are shown. Scale bar = 250 µm. (D) Total RNA was extracted and mRNA for mouse CXCL1 and GAPDH were evaluated by real-time PCR analysis. Data were normalized using GAPDH transcripts. This graph shows the results of three independent experiments.

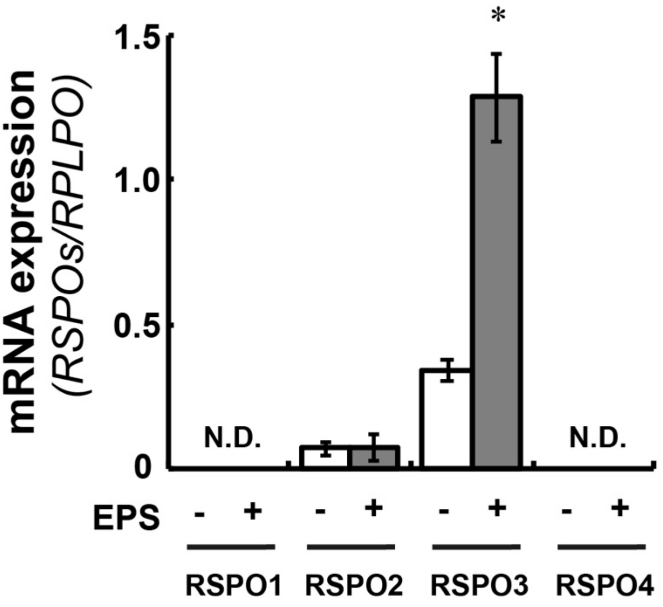

Figure S2. Effects of EPS on RSPO1, 2, 3, and 4 mRNA expressions in human myotubes cultured in the insert chamber

After 7-8 days of differentiation, the insert chambers were placed in an 8-well plate set on a C-Pace, and EPS (1 Hz, 4-ms, 20V/25 mm) was applied to the differentiated myotubes for a total of 24 h, as described in the Methods. Total RNA was extracted and subjected to real-time PCR analysis to determine mRNA levels for human RSPO1, 2, 3, and 4, as well as RPLP0. Data normalized using RPLP0 transcripts were averaged over 3 independent experiments (*P<0.05).

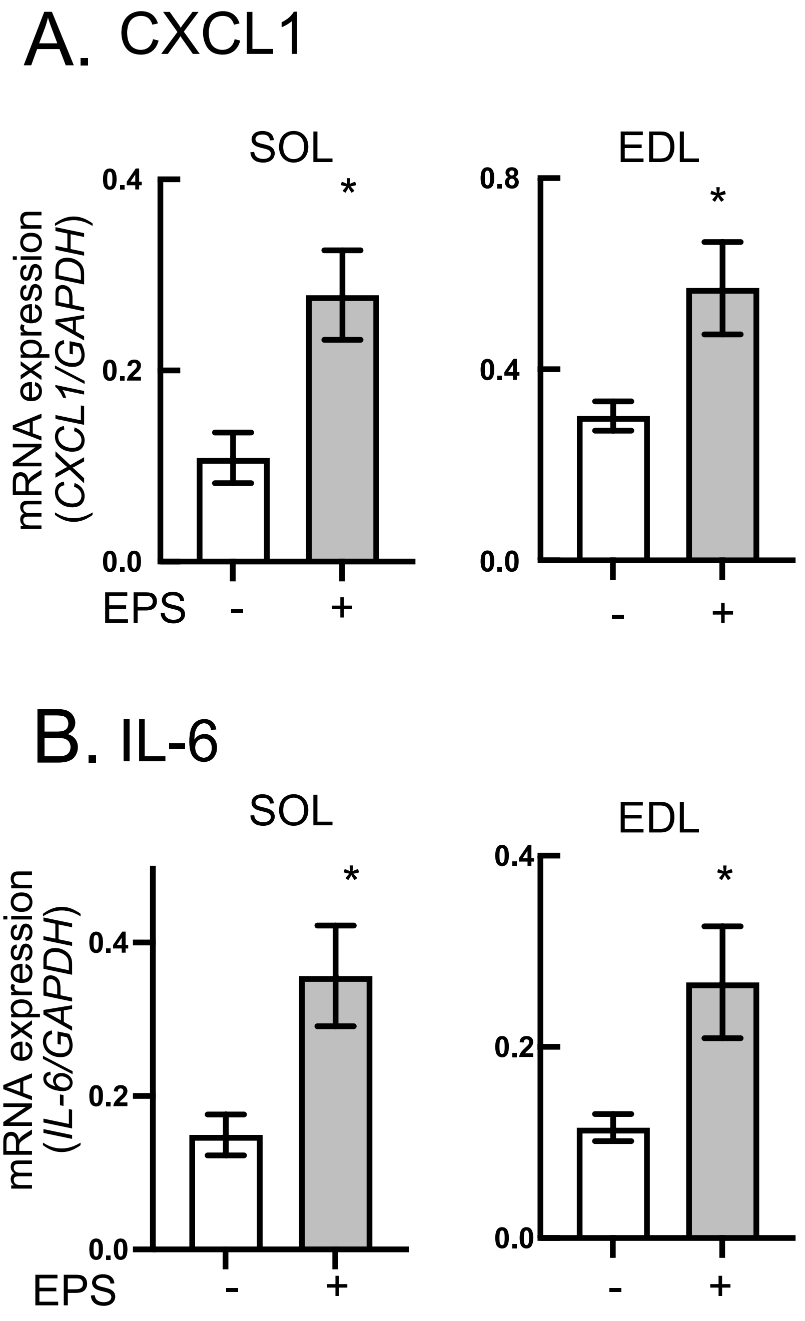

Figure S3. Upregulations of CXCL1 and IL-6 mRNAs in response to in situ muscle contraction in mice

After the in situ muscle contraction via sciatic nerve-mediated EPS treatment as described in the Methods, soleus (SOL) and extensor digitorum longus (EDL) muscles were obtained and subjected to RT-PCR analysis to determine mRNA levels

for CXCL1 (A) and IL-6 (B). Data were normalized using mouse GAPDH transcripts and compared to those of the muscle tissues obtained from sham-operated control (opposite side) legs (n = 9; *P<0.05).
